# Low serum docosahexaenoic acid is associated with progression of coronary atherosclerosis in statin-treated patients with diabetes mellitus: results of the treatment with statin on atheroma regression evaluated by intravascular ultrasound with virtual histology (TRUTH) study

**DOI:** 10.1186/1475-2840-13-13

**Published:** 2014-01-13

**Authors:** Tsuyoshi Nozue, Shingo Yamamoto, Shinichi Tohyama, Kazuki Fukui, Shigeo Umezawa, Yuko Onishi, Tomoyuki Kunishima, Akira Sato, Toshihiro Nozato, Shogo Miyake, Youichi Takeyama, Yoshihiro Morino, Takao Yamauchi, Toshiya Muramatsu, Kiyoshi Hibi, Mitsuyasu Terashima, Ichiro Michishita

**Affiliations:** 1 Division of Cardiology, Department of Internal Medicine, Yokohama Sakae Kyosai Hospital, Federation of National Public Service Personnel Mutual Associations, 132 Katsura-cho, Sakae-ku, Yokohama 247-8581, Japan; 2 Department of Cardiology, Tsurumi Nishiguchi Hospital, Yokohama Japan; 3 Department of Cardiology, Yokohama Seamen’s Insurance Hospital, Yokohama Japan; 4 Department of Cardiology, Kanagawa Cardiovascular and Respiratory Center, Yokohama Japan; 5 Department of Cardiology, Hiratsuka Kyosai Hospital, Hiratsuka, Japan; 6 Fourth Department of Internal Medicine, Mizonokuchi Hospital, Teikyo University School of Medicine, Kawasaki Japan; 7 Cardiovascular Center, Yokosuka Kyosai Hospital, Yokosuka Japan; 8 Department of Cardiology, National Hospital Organization, Disaster Medical Center, Tokyo Japan; 9 Department of Cardiology, Ebina General Hospital, Ebina, Japan; 10 Division of Cardiology, Showa University Fujigaoka Rehabilitation Hospital, Yokohama, Japan; 11 Department of Cardiology, Tokai University School of Medicine, Isehara, Japan; 12 Department of Cardiology, Tokyo Women’s Medical University, Tokyo, Japan; 13 Department of Cardiology, Saiseikai Yokohama City Eastern Hospital, Yokohama, Japan; 14 Division of Cardiology, Yokohama City University Medical Center, Yokohama, Japan; 15 Cardiovascular Imaging Center, Toyohashi, Japan

**Keywords:** Atheroma, Coronary atherosclerosis, Diabetes mellitus, Statin, Virtual histology intravascular ultrasound

## Abstract

**Background:**

Diabetes mellitus (DM) accelerates plaque progression despite the use of statin therapy. The purpose of the present study was to evaluate the determinants of atheroma progression in statin-treated patients with DM.

**Methods:**

Coronary atherosclerosis in nonculprit lesions in a vessel undergoing percutaneous coronary intervention (PCI) was evaluated using virtual histology intravascular ultrasound. The study included 50 patients with DM who had been taking statin therapy for 8 months at the time of PCI.

**Results:**

Twenty-six patients (52%) showed atheroma progression (progressors) and the remaining 24 patients (48%) showed atheroma regression (regressors) after 8 months of follow-up. Fewer progressors than regressors received intensive lipid-lowering therapy with pitavastatin (31% vs. 50%, p = 0.17) and the frequency of insulin use was higher in progressors (31% vs. 13%, p = 0.18). However, neither of these differences reached statistical significance. Risk factor control at baseline and at the 8-month follow-up did not differ between the 2 groups except for serum levels of eicosapentaenoic acid (EPA) and docosahexaenoic acid (DHA). Univariate regression analysis showed that serum EPA (r = -0.317, p = 0.03) and DHA (r = -0.353, p = 0.02) negatively correlated with atheroma progression. Multivariate stepwise regression analysis showed that low serum DHA and pravastatin use were significant independent predictors for atheroma progression during statin therapy (DHA: β = -0.414, type of statin: β = -0.287, p = 0.001).

**Conclusions:**

Low serum DHA is associated with progression of coronary atherosclerosis in statin-treated patients with DM.

**Trial registration:**

UMIN Clinical Trials Registry, UMIN ID: C000000311.

## Background

Patients with diabetes mellitus (DM) are at high risk for developing coronary artery disease (CAD) [1]. Recent clinical trials using grayscale intravascular ultrasound (IVUS) have shown that intensive lipid-lowering therapy with statins results in the regression of coronary artery plaques [2] and reduces the risk of coronary events [3,4]. However, cardiovascular events have not been reduced by statin therapy in patients with DM [5]. Furthermore, patients with DM have significantly more coronary events compared to those without DM who are receiving statin therapy [6]. This occurs because DM accelerates plaque progression despite the use of statin therapy [7-9]. In view of this evidence, it is important to identify factors that are associated with plaque progression despite the use of statin therapy, particularly in patients with DM. Therefore, in this study, we evaluated the determinants of atheroma progression in statin-treated patients with DM.

## Methods

### Study design

Our study is a post-hoc subanalysis of the Treatment with Statin on Atheroma Regression Evaluated by IVUS with Virtual Histology (TRUTH) study. The TRUTH study was a prospective, open-labeled, randomized, multicenter trial performed at 11 Japanese centers to evaluate the effect of 8 months of treatment with pitavastatin versus pravastatin on coronary atherosclerosis using virtual histology (VH)-IVUS [10]. In brief, 164 patients with angina pectoris were randomized to either pitavastatin (4 mg/day, intensive lipid-lowering therapy) or pravastatin (20 mg/day, moderate lipid-lowering therapy) after successful percutaneous coronary intervention (PCI) under VH-IVUS guidance. Follow-up IVUS examination was performed after 8 months of statin therapy.

The inclusion criteria were having DM and analyzable IVUS data obtained at the time of PCI and at the 8-month follow-up. Diagnosis of DM was defined as follows: (1) fasting plasma glucose level ≥ 126 mg/dl, and/or (2) random plasma glucose level ≥ 200 mg/dl, and/or (3) hemoglobin A1c (HbA1c) level ≥ 6.5%, and/or (4) treatment with either oral hypoglycemic agents or insulin [11]. Of the original 164 study patients, 114 did not meet inclusion criteria, leaving 50 patients who were ultimately included in the study. These patients were divided into 2 groups: progressors and regressors. A progressor was defined as a patient whose change in plaque volume between the 8-month follow-up and baseline was ≥0. A regressors was defined as a patient whose change in plaque volume between the 8-month follow-up and baseline was 〈0.

Using data from the TRUTH trial, we compared the clinical characteristics, risk factor control, and grayscale and VH-IVUS parameters between the progressors and regressors. The TRUTH trial was conducted in accordance with the Declaration of Helsinki and with the approval of the institutional ethical committees of the 11 participating institutions. Each patient enrolled in this study provided written informed consent.

### IVUS examination and analysis

The details of the IVUS procedure and examination are documented [10]. In brief, after PCI of the culprit lesion, IVUS examination was performed on angiographic lesions with 〈50% lumen narrowing on both the distal and proximal sides of the culprit lesion. An Eagle Eye Gold IVUS catheter (Volcano Corporation, San Diego, California) was used with a motorized pullback device to withdraw the transducer at 0.5 mm/s. During pullback, grayscale IVUS was recorded, and raw radiofrequency data were captured at the top of the R-wave by using a commercially available IVUS console (IVG3; Volcano Corporation). After 8 months of statin therapy, the IVUS examination was repeated in the same coronary artery using the same type of IVUS catheter used at baseline.

All baseline and follow-up IVUS core laboratory analyses were performed by an independent and experienced investigator (M. T.) in a blinded manner. Before IVUS analysis, baseline and follow-up IVUS images were reviewed side-by-side on a display, and the distal and proximal ends of the target segment were identified by reproducible anatomic landmarks such as the side-branch, vein, and stent edge. Plaques close to the PCI site (within 5 mm) were excluded. Manual contour detection of both the lumen and the external elastic membrane (EEM) was performed for each frame. Quantitative IVUS grayscale analysis was performed according to the guidelines of the American College of Cardiology and European Society of Cardiology [12]. All volumetric data were divided by lesion length to obtain a volume index. VH-IVUS data analysis was based on grayscale border contour calculation, and relative and absolute amounts of different coronary artery plaque components were measured by using IVUSLab version 2.2 (Volcano Corporation).

### Laboratory determinations

Blood examinations for lipid levels were performed at baseline and at the 8-month follow-up. Levels of serum lipids, apolipoproteins, and high-sensitivity C-reactive protein (hs-CRP) were measured at a central clinical laboratory (SRL, Inc., Tokyo). The serum levels of eicosapentaenoic acid (EPA), docosahexaenoic acid (DHA), and arachidonic acid (AA) at baseline and at the 8-month follow-up were measured by a central laboratory (BML, Inc., Kawagoe).

### Statistical analysis

Statistical analyses were performed by using StatView version 5.0 (SAS Institute, Cary, North Carolina). The results were expressed as mean ± SD. Differences in continuous variables between the 2 groups were compared by using an unpaired t-test when the variables showed a normal distribution and the Mann–Whitney U-test when the variables were not normally distributed. Differences in continuous variables within each group were compared by using paired t-tests when the variables showed a normal distribution and Wilcoxon’s signed-rank sum test when the variables were not normally distributed. Categorical variables between the 2 groups were compared by using the chi-square test or Fisher’s exact test. Univariate regression analysis was performed to determine the predictors of atheroma progression during statin therapy. Multivariate stepwise regression analysis was performed among variables with a p value 〈 0.2 in univariate analysis (hypertension, high-density lipoprotein cholesterol, apolipoprotein AI and B, EPA, DHA, and AA). Statistical significance was set at p 〈 0.05.

## Results

### Patient characteristics and risk factor control

Baseline characteristics of subjects are listed in Table 1. Twenty-six patients (52%) were included in the progressor group, and the remaining 24 (48%) were included in the regressor group. There were no significant differences in baseline characteristics between the 2 groups. Fewer progressors than regressors received intensive lipid-lowering therapy with pitavastatin (31% vs. 50%, p = 0.17) and the frequency of insulin use was higher in progressors (31% vs. 13%, p = 0.18). However, neither of these differences reached statistically significant.

**Table 1:** Baseline characteristics of subjects

	**Progressors (n=26)**	**Regressors (n=24)**	**p value**
Age (years)	69 ± 10	66 ± 10	0.28
Men	22 (85%)	17 (71%)	0.31
Body mass index (kg/m^2^)	24.3 ± 2.5	26.5 ± 4.8	0.06
eGFR (ml/min/1.73m^2^)	63.4 ± 14.1	63.7 ± 15.9	0.94
Hypertension	21 (81%)	14 (58%)	0.12
Smoker	8 (31%)	3 (13%)	0.28
Pitavastatin/Pravastatin	8 (31%)/18 (69%)	12 (50%)/12 (50%)	0.17
Stable AP/Unstable AP	18 (69%)/8 (31%)	17 (71%)/7 (29%)	0.9
Target coronary artery			0.84
Left anterior descending	12 (46%)	13 (54%)	
Left circumflex	1 (4%)	1 (4%)	
Right	13 (50%)	10 (42%)	
Types of stent			>0.99
Bare metal stent	3 (12%)	3 (12.5%)	
Drug-eluting stent	23 (88%)	21 (87.5%)	
Medications			
Aspirin	26 (100%)	23 (96%)	0.48
Thienopyridines	26 (100%)	24 (100%)	-
ACE inhibitors or ARBs	17 (65%)	12 (50%)	0.27
β Blockers	1 (4%)	2 (8%)	0.6
Calcium channel blockers	16 (62%)	11 (46%)	0.27
Insulin	8 (31%)	3 (13%)	0.18
Thiazolidine	3 (12%)	3 (13%)	>0.99
Follow-up duration (days)	236 ± 32	231 ± 28	0.58

Data are expressed as the mean ± SD or as number (%).

*eGFR*, estimated glomerular filtration rate; *AP*, angina pectoris; *ACE*, angiotensin-converting enzyme; *ARB*, angiotensin receptor blocker.

Risk factor control at baseline and at the 8-month follow-up is listed in Table 2. Serum levels of total cholesterol, low-density lipoprotein cholesterol (LDL-C), triglycerides, high-density lipoprotein cholesterol, hs-CRP, oxidized LDL, lipoprotein(a), and small dense LDL at baseline and at the 8-month follow-up did not differ between the 2 groups. Furthermore, there were no significant differences in plasma glucose and HbA1c levels between the 2 groups. However, serum levels of EPA and DHA at the 8-month follow-up were significantly lower in progressors than in regressors. Serum AA levels also tended to be lower in progressors but the difference did not reach statistical significance. EPA/AA, DHA/AA, and EPA+DHA/AA ratios did not differ between the 2 groups.

**Table 2:** Risk factor control at baseline and at follow-up

	**At baseline**			**At 8-month follow-up**		
	**Progressors (n=26)**	**Regressors (n=24)**	**p value**	**Progressors (n=26)**	**Regressors (n=24)**	**p value**
TC (mg/dl)	203 ± 44	218 ± 31	0.18	153 ± 31****	165 ± 22***	0.12
LDL-C (mg/dl)	133 ± 36	140 ± 29	0.49	85 ± 27****	88 ± 21***	0.67
Triglycerides (mg/dl)	127 ± 55	157 ± 96	0.18	114 ± 53	141 ± 85	0.18
HDL-C (mg/dl)	44 ± 9	46 ± 9	0.6	46 ± 11	51 ± 11*	0.1
Apolipoprotein AI (mg/dl)	114 ± 19	117 ± 16	0.55	122 ± 25	134 ± 25**	0.1
Apolipoprotein B (mg/dl)	102 ± 25	114 ± 21	0.08	70 ± 19****	79 ± 15****	0.1
Hs-CRP (ng/ml)	7023 ± 14355	7298 ± 9171	0.94	2856 ± 5923	2853 ± 5630*	0.99
Oxidized LDL (U/ml)	9 ± 4	12 ± 10	0.18	8 ± 3	9 ± 4*	0.6
Lipoprotein(a) (mg/dl)	16 ± 13	18 ± 14	0.63	19 ± 18	24 ± 28	0.46
Small dense LDL (mg/dl)	26 ± 16	31 ± 16	0.32	18 ± 10***	21 ± 11*	0.32
EPA (μg/ml)	53.5 ± 28.2	71.9 ±39.2	0.08	54.9 ± 22.9	74.8 ± 36.6	0.03
DHA (μg/ml)	117.2 ± 38.3	149.3 ± 62.8	0.04	105.1 ± 31.8	135.9 ± 50.1	0.02
AA (μg/ml)	128.4 ± 35.7	144.2 ± 30.3	0.12	133.2 ± 38.2	155.8 ± 41.9	0.07
EPA/AA	0.43 ± 0.20	0.52 ± 0.34	0.26	0.44 ± 0.22	0.53 ± 0.35	0.33
DHA/AA	0.94 ± 0.28	1.04 ± 0.32	0.23	0.83 ± 0.30*	0.90 ± 0.29*	0.47
EPA + DHA/AA	1.36 ± 0.46	1.57 ± 0.58	0.19	1.28 ± 0.50	1.43 ± 0.56	0.34
Glucose (mg/dl)	135 ± 56	123 ± 39	0.44	122 ± 34	124 ± 48	0.85
HbA1c (%)	7.2 ± 1.0	7.0 ± 0.8	0.38	6.9 ± 0.9	7.0 ± 0.6	0.77
Systolic blood pressure (mmHg)	136 ± 17	134 ± 23	0.83	134 ± 20	133 ± 23	0.88
Diastolic blood pressure (mmHg)	76 ± 12	75 ± 12	0.75	73 ± 13	75 ± 12	0.56
Heart rate (beats/min)	71 ± 12	71 ± 11	0.98	72 ± 11	70 ± 15	0.5

Data are expressed as mean ± SD. *p 〈 0.05, **p 〈 0.01, ***p 〈 0.001, ****p 〈 0.0001 compared with value at baseline.

*TC*, total cholesterol; *LDL-C*, low-density lipoprotein cholesterol; *HDL-C*, high-density lipoprotein cholesterol; *Hs-CRP*, high-sensitivity C-reactive protein; *EPA*, eicosapentaenoic acid; *DHA*, docosahexaenoic acid; AA, arachidonic acid; HbA1c, hemoglobin A1c.

### Grayscale and VH-IVUS analysis

The parameters evaluated by using grayscale and VH-IVUS are shown in Table 3. There were significant differences in percentage change of EEM volume index (progressors: 0.2% vs. regressors: -2.7%, p = 0.01) and lumen volume index (progressors: -6.6% vs. regressors: 2.4%, p = 0.0002) between the 2 groups. Furthermore, necrotic core (progressors: 1.01 mm^3^/mm vs. regressors: 0.54 mm^3^/mm, p = 0.005) and dense calcium (progressors: 0.64 mm^3^/mm vs. regressors: 0.34 mm^3^/mm, p = 0.02) plaque volume at baseline were significantly greater in progressors than in regressors.

**Table 3:** Parameters evaluated by using grayscale and virtual histology intravascular ultrasound

	**At baseline**			**At 8-month follow-up**		
	**Progressors (n=26)**	**Regressors (n=24)**	**p value**	**Progressors (n=26)**	**Regressors (n=24)**	**p value**
EEM volume index (mm^3^/mm)	16.97 ± 5.65	16.47 ± 6.17	0.77	17.01 ± 5.80	16.03 ± 6.04***	0.56
% change				0.2 ± 4.5	-2.7 ± 3.0	0.01
Plaque volume index (mm^3^/mm)	9.59 ± 3.84	9.48 ± 4.06	0.93	10.11 ± 3.86****	8.83 ± 3.61***	0.23
% change				6.3 ± 5.9	-6.4 ± 4.5	0.0001
Lumen volume index (mm^3^/mm)	7.38 ± 2.82	6.99 ± 2.37	0.6	6.89 ± 2.69**	7.20 ± 2.73	0.69
% change				-6.6 ± 9.0	2.4 ± 6.8	0.0002
Percent atheroma volume (%)	56.2 ± 8.6	57.0 ± 6.1	0.69	59.3 ± 7.8****	54.9 ± 6.4***	0.03
Nominal change (%)				3.2 ± 2.8	-2.2 ± 2.4	0.0001
Fibrous volume index (mm^3^/mm)	3.52 ± 2.29	3.72 ± 2.18	0.75	3.92 ± 1.98*	3.11 ± 1.80****	0.14
Change (mm^3^/mm)				0.40 ± 0.85	-0.61 ± 0.53	0.0001
Fibrofatty volume index (mm^3^/mm)	1.08 ± 0.77	1.42 ± 1.44	0.29	0.93 ± 0.84	1.06 ± 0.93	0.62
Change (mm^3^/mm)				-0.15 ± 0.57	-0.37 ± 0.94	0.31
Necrotic core volume index (mm^3^/mm)	1.01 ± 0.73	0.54 ± 0.30	0.005	1.10 ± 0.69	0.74 ± 0.49*	0.04
Change (mm^3^/mm)				0.09 ± 0.64	0.20 ± 0.38	0.47
Dense calcium volume index (mm^3^/mm)	0.64 ± 0.60	0.34 ± 0.18	0.02	0.75 ± 0.61	0.48 ± 0.43	0.08
Change (mm^3^/mm)				0.11 ± 0.34	0.13 ± 0.35	0.77
Average length (mm)	27.5 ± 16.3	26.1 ± 13.0	0.74	27.6 ± 17.8	26.3 ± 12.7	0.77

Data are expressed as mean ± SD. *p 〈 0.05, **p 〈 0.01, ***p 〈 0.001, ****p 〈 0.0001 compared with value at baseline.

*EEM*, external elastic membrane.

### Predictor of atheroma progression during statin therapy

Univariate regression analysis showed that serum EPA (r = -0.317, p = 0.03) and DHA (r = -0.353, p = 0.02) negatively correlated with atheroma progression during statin therapy and presence of hypertension (r = 0.245, p = 0.09) and serum AA (r = -0.277, p = 0.07) trended in that direction (Table 4). Multivariate stepwise regression analysis showed that low serum DHA and pravastatin use were significant independent predictors of atheroma progression during statin therapy (DHA: β = -0.414, type of statin: β = -0.287, p = 0.001; Table 5).

**Table 4:** Univariate predictors of atheroma progression during statin therapy

	**r**	**p value**
Age	0.156	0.28
Gender	0.166	0.25
Coronary artery disease status	0.017	0.9
Hypertension	0.245	0.09
Smoking	0.220	0.12
Type of statin	-0.196	0.17
TC	-0.224	0.12
LDL-C	-0.061	0.67
Triglycerides	-0.192	0.18
HDL-C	-0.237	0.1
Apolipoprotein AI	-0.241	0.1
Apolipoprotein B	-0.239	0.1
Hs-CRP	2.576	0.99
Oxidized LDL	-0.080	0.6
Lipoprotein(a)	-0.109	0.46
Small dense LDL	-0.151	0.32
EPA	-0.317	0.03
DHA	-0.353	0.02
AA	-0.277	0.07
EPA/AA	-0.149	0.33
DHA/AA	-0.111	0.47
EPA + DHA/AA	-0.145	0.34
Glucose	-0.028	0.85
HbA1c	-0.050	0.77
Insulin	0.220	0.12
eGFR	0.038	0.79

Male gender, unstable angina pectoris, hypertension, smoking, pitavastatin, and insulin use were assigned a value of 1. Female gender, stable angina pectoris, normotension, nonsmoking, pravastatin and no insulin use were assigned a value of 0.

Abbreviations as in Table 2.

**Table 5:** Multivariate stepwise regression coefficients and F values for atheroma progression during statin therapy

**Step variables**	**β**	**SE**	**F value**
1. DHA	-0.414	0.002	8.633
2. Type of statin	-0.287	0.142	4.134

Multivariate stepwise regression analysis was performed using 12 variables: hypertension, smoking, type of statin, insulin use, TC, TG, HDL-C, apolipoprotein AI, apolipoprotein B, EPA, DHA, and AA. Hypertension, smoking, pitavastatin, and insulin use was assigned a value of 1. Normotension, nonsmoking, pravastatin, and no insulin use was assigned a value of 0.

## Discussion

The main findings of our study are: (1) low serum DHA and pravastatin use were associated with atheroma progression in statin-treated patients with DM; (2) plasma glucose and HbA1c levels did not correlate with plaque progression or regression during statin therapy; and (3) volume of the necrotic core component at baseline was associated with atheroma progression during statin therapy.

### Residual risk of cardiovascular events during statin therapy

DM is accompanied by extensive atherosclerosis deposition despite the use of medications [8], and statins are significantly less effective at inducing regression of coronary artery plaque in patients with DM [7,9]. Not all patients show atheroma regression and enhanced prevention of cardiovascular events while taking statin therapy, particularly diabetic patients. Serum LDL-C levels at the 8-month follow-up did not differ between the progressors and regressors, whereas low serum DHA was associated with progression of coronary atherosclerosis during statin therapy. On the basis of this study’s results, the residual risk of cardiovascular events during statin therapy can be explained in part by n-3 polyunsaturated fatty acids (PUFAs). Indeed, highly purified EPA is very effective in decreasing the incidence of CAD in patients with impaired glucose metabolism [13].

### Surrogate marker of PUFAs

Plasma levels of EPA and DHA have been associated with reduced risk of sudden death [14] and fatal ischemic heart disease [15]. However, the parameter that is the best surrogate marker of PUFAs for cardiovascular events remains unclear. Several authors tended to explain the PUFAs effects in terms of a balance between n-3 to n-6 PUFAs, rather than the absolute amount of each single molecule. Domei et al. reported that lower EPA/AA ratio, but not EPA, DHA, or DHA/AA ratio, was associated with higher incidence of cardiac events [16]. However, a recent cohort study reported that a lower EPA/AA ratio was associated with a greater risk of cardiovascular events in patients with higher hs-CRP levels, but no clear association was observed in those with lower hs-CRP levels [17]. In addition, the other cohort study reported that plasma EPA and DHA, but not AA, were inversely associated with cardiovascular events [18]. Thus, although serum EPA/AA ratio is one of the useful markers for the risk of cardiovascular events, this ratio does not apply to all patients. Heterogeneity in population characteristics, background dietary fish consumption, and length of follow-up may each contribute to these inconsistent findings from different study designs. The present study found a low DHA to be associated with progression of coronary atherosclerosis, indicating that low DHA could be one of biomarkers for progression of coronary atherosclerosis. Furthermore, our results suggest that EPA and DHA may have different effects on coronary atherosclerosis, particularly in patients with DM. This may be because of the enhanced peroxisomal β-oxidation and reduced n-3 PUFAs content in diabetic heart [19].

### PUFAs and cardiovascular events

The intake of fish is associated with a lower risk of cardiovascular disease [20,21]. Several mechanisms may explain the favorable association between n-3 PUFAs and cardiovascular risk. One of these mechanisms is that n-3 PUFAs beneficially alter the cardiovascular risk factor profile [22,23]. In experimental studies, higher n-3 PUFAs levels alter cell membrane fluidity and receptor responses, regulate gene transcription, and serve as metabolic precursors to potent anti-inflammatory molecules [24]. These molecular effects may underline their systemic and cardiac benefits such as inflammatory responses, autonomic control, vascular and cardiac hemodynamics, endothelial function, blood lipids, and possibly thrombosis [24,25]. Data from intervention studies have also identified a lower risk of cardiovascular events associated with purified EPA (1800 mg/day) [26] as well as EPA+DHA (1 g/day) [27]. Pharmacological differences between EPA and DHA have become clear in recent years, but it is still unclear as to which of these n-3 PUFAs has a strong antiatherogenic effect. A clinical trial reported that DHA, but not EPA, has been associated with reduced progression of coronary atherosclerosis [28]. Two additional studies reported that DHA, but not EPA, showed a significant inverse association with intima-media thickness [29,30]. The Japan Eicosapentaenoic Acid Lipid Intervention Study (JELIS) showed that highly purified EPA reduced nonfatal CAD [26]. Although the JELIS study did not report the serum DHA level during trial, the reduction of nonfatal CAD is more likely to be mediated through EPA rather than DHA. In the present study, high serum EPA was associated with atheroma regression in univariate analysis, whereas this correlation did not observe in multivariate analysis. Therefore, our results may suggest that DHA has a more potent antiatherogenic effect than EPA.

### Plasma glucose control and cardiovascular events

Although intensive plasma glucose control with sulfonylureas or insulin does not reduce macrovascular disease [31], pioglitazone reduces the composite of all-cause mortality, non-fatal myocardial infarction, and stroke in patients with DM [32]. Furthermore, pioglitazone prevents progression of coronary atherosclerosis [33] and stabilizes coronary atherosclerosis [34], suggesting that this medication may have beneficial effect on coronary artery plaques. Plasma glucose and HbA1c levels did not correlate with atheroma progression or regression in this study. This might be due to the fact that antidiabetic medications were not fixed during the study period.

### Statin therapy for secondary prevention of cardiovascular events

Compared with moderate lipid-lowering therapy, intensive lipid-lowering therapy with a statin provides a significantly greater reduction of coronary events [3,4]. Indeed, atorvastatin 80 mg significantly reduced the rate of cardiovascular events by 25% compared with atorvastatin 10 mg in patients with DM [35]. This indicates that intensive LDL-C control is necessary for secondary prevention of CAD, particularly in patients with DM. The American Heart Association and American College of Cardiology offer an optimal LDL-C goal of 〈 70 mg/dl for patients with a very high risk of CAD [36]. In Japan, a provisional therapeutic goal of LDL-C with statins is 〈 75 mg/dl in patients with the highest risk conditions (those with higher baseline LDL-C level, DM, or chronic kidney disease) [37]. However, in our study, LDL-C levels at the 8-month follow-up were 85 mg/dl in progressors and 88 mg/dl in regressors. More vigorous reduction in LDL-C levels would be necessary to produce regression of coronary atherosclerosis in patients with DM as previously reported [38]. Serum PUFAs have less impact on coronary atherosclerosis in patients treated with strong statin because a greater reduction in LDL-C mainly affects coronary atherosclerosis [39]. The results of this study suggest that diabetic patients should be treated with strong statin at intensive lipid-lowering dose rather than weak statin at moderate lipid-lowering doses for secondary prevention of CAD.

We also observed that necrotic core and calcified plaque volume at baseline was significantly greater in progressors than in regressors. This may reflect ongoing, irreversible plaque progression once the disease has reached a critical point. It may also suggest that treatment with delayed statin therapy would potentially be a risk for atheroma progression and lead to future cardiovascular events. The findings from previous trial support a strategy of the early initiation of an aggressive LDL-C lowering to prevent death and major cardiovascular events [40].

The present study had several limitations. First, it is a post-hoc subanalysis of the TRUTH trial. Second, diagnosis of DM was made by the attending physician and a glucose tolerance test was not conducted for all potential study subjects. Patients with only impaired glucose tolerance or borderline diabetes were not diagnosed as having DM. Third, antidiabetic and antihypertensive medications were not fixed during the study period. Changes in medications may have affected the results of this study. Fourth, patients were not prohibited from making lifestyle changes. Changes in PUFA composition of the diet likely reflect changes in serum PUFA levels. Finally, the statistical power was insufficient to characterize the determinants of atheroma progression because of the small number of patients. For these reasons, a prospective, randomized, multicenter study is necessary to compare intensive versus moderate lipid-lowering statin therapy on coronary artery plaque volume in patients with DM.

## Conclusions

We found low serum DHA was associated with progression of coronary atherosclerosis in statin-treated patients with DM. Intensive lipid-lowering therapy with strong statin or n-3 PUFAs add on statin therapy is necessary to produce atheroma regression for secondary prevention of CAD, particularly in patients with DM.

## Abbreviations

AA: Arachidonic acid; CAD: Coronary artery disease; DHA: Docosahexaenoic acid; DM: Diabetes mellitus; EEM: External elastic membrane; EPA: Eicosapentaenoic acid; HbA1c: Hemoglobin A1c; hs-CRP: High-sensitivity C-reactive protein; IVUS: Intravascular ultrasound; LDL-C: Low-density lipoprotein cholesterol; PCI: Percutaneous coronary intervention; PUFA: Polyunsaturated fatty acid; TRUTH: Treatment with statin on atheroma regression evaluated by intravascular ultrasound with virtual histology; VH: Virtual histology.

## Competing interests

The authors declare that they have no competing interest.

## Authors’ contributions

TN contributed to the study design, analysis and interpretation of data, and manuscript preparation. SY, ST, KF, SU, YO, TK, AS, TN, SM, YT, YM, TY and TM contributed to acquisition of data. KH contributed to the study design. MT contributed to the analysis of the data. IM contributed to the study design and managed the study. All authors read and approved the final manuscript.
